# Characterization of Neoagarooligosaccharide Hydrolase *Bp*GH117 from a Human Gut Bacterium *Bacteroides plebeius*

**DOI:** 10.3390/md19050271

**Published:** 2021-05-13

**Authors:** Yerin Jin, Sora Yu, Dong Hyun Kim, Eun Ju Yun, Kyoung Heon Kim

**Affiliations:** Department of Biotechnology, Graduate School, Korea University, Seoul 02841, Korea; dpfls96@korea.ac.kr (Y.J.); sora90715@korea.ac.kr (S.Y.); goodsoul4u@korea.ac.kr (D.H.K.); jdjddcld@korea.ac.kr (E.J.Y.)

**Keywords:** α-neoagarooligosaccharide hydrolase, exo-acting 3,6-anhydro-α-(1,3)-L-galactosidase, *Bp*GH117, 3,6-anhydro-L-galactose, human gut bacterium, *Bacteroides plebeius*, agarose

## Abstract

α-Neoagarobiose (NAB)/neoagarooligosaccharide (NAO) hydrolase plays an important role as an exo-acting 3,6-anhydro-α-(1,3)-L-galactosidase in agarose utilization. Agarose is an abundant polysaccharide found in red seaweeds, comprising 3,6-anhydro-L-galactose (AHG) and D-galactose residues. Unlike agarose degradation, which has been reported in marine microbes, recent metagenomic analysis of *Bacteroides plebeius*, a human gut bacterium, revealed the presence of genes encoding enzymes involved in agarose degradation, including α-NAB/NAO hydrolase. Among the agarolytic enzymes, *Bp*GH117 has been partially characterized. Here, we characterized the exo-acting α-NAB/NAO hydrolase *Bp*GH117, originating from *B. plebeius*. The optimal temperature and pH for His-tagged *Bp*GH117 activity were 35 °C and 9.0, respectively, indicative of its unique origin. His-tagged *Bp*GH117 was thermostable up to 35 °C, and the enzyme activity was maintained at 80% of the initial activity at a pre-incubation temperature of 40 °C for 120 min. *K_m_* and *V_max_* values for NAB were 30.22 mM and 54.84 U/mg, respectively, and *k_cat_/K_m_* was 2.65 s^−1^ mM^−1^. These results suggest that His-tagged *Bp*GH117 can be used for producing bioactive products such as AHG and agarotriose from agarose efficiently.

## 1. Introduction

Diet plays an important role in gut microbiome formation, and dietary changes show transient but significant microbial population changes in the gut [1]. Among various dietary components, non-digestible carbohydrates such as resistant starch and fiber cannot be decomposed in the small intestine. Instead, when non-digestible carbohydrates reach the large intestine, they are utilized by resident microorganisms. Therefore, diet can change intestinal microflora and consequently affect overall host health [2,3,4].

Marine red macroalgae, one of the representative non-digestible diets, especially in East Asia, has received much attention as an important food resource [5,6]. Most enzymes required to degrade red macroalgae are known to originate from marine microorganisms [7,8,9]. However, recent studies have revealed that human gut microbes also carry genes encoding Carbohydrate-Active enZymes (CAZymes), which can hydrolyze marine polysaccharides, including agarose [10,11,12]. Additionally, it was suggested that the genes encoding CAZymes involved in agarose degradation have been transferred from the marine bacterium *Zobellia galactanivorans* to the human gut bacterium *Bacteroides plebeius*, which was isolated from the microbiota of Japanese individuals [10,13]. This implies that human gut microbes may help humans utilize red seaweeds that cannot be degraded by the innate enzymes found in humans.

Agar, a major polysaccharide in the cell wall of marine red macroalgae, comprises agarose and porphyran [13,14]. Agarose, which occupies 70–80% of agar, is a neutral and linear polysaccharide composed of alternating 3,6-anhydro-L-galactose (AHG) and D-galactose by α-1,3- and β-1,4-glycosidic linkages [15]. Agarases have been extensively studied for the cleavage of the β-1,4 bonds in agarose [16,17]. However, little is known about the biochemical characteristics of 3,6-anhydro-α-(1,3)-L-galactosidases, including α-neoagarobiose hydrolase (α-NABH) and α-neoagarooligosaccharide hydrolase (α-NAOH) belonging to the glycoside hydrolase 117 family (GH117), compared to agarases [18]. Since all agarolytic bacteria contain at least one conserved GH117 enzyme, GH117 appears to be the major evolutionary solution for cleaving α-1,3 glycosidic bonds in agarose [19]. This suggests that GH117 enzymes are important for polysaccharide utilization in agarolytic bacteria.

*B. plebeius* was shown to have an exo-acting 3,6-anhydro-α-(1,3)-L-galactosidase, *Bp*GH117, which belongs to GH117 and removes AHG from the non-reducing end of neoagarooligosaccharide (NAO) of agarose [19]. *Bp*GH117 decomposes neoagarotetraose (NeoDP4) into AHG and agarotriose (AgaDP3), and also neoagarobiose (NeoDP2) into AHG and galactose [19]. Lately, AgaDP3 has been found to have various health-benefiting effects. AgaDP3 is suggested to be a prebiotic since it is utilized by probiotic strains *Bifidobacterium infantis* and *Bifidobacterium adolescentis* [11]. Additionally, in vitro anti-colon cancer activity of AgaDP3 has been revealed recently [20]. In addition, AHG has been shown to have skin whitening, anticariogenic, and anti-inflammatory effects [21,22]. Although *Bp*GH117 has the potential to be used to produce high value-added products from agarose, it has only been partially characterized. Enzymatic properties such as optimal pH and temperature, and kinetic parameters of His-tagged *Bp*GH117, remain unknown.

In this study, we characterized His-tagged *Bp*GH117 originating from a human gut bacterium, *B. plebeius*. The characteristics of His-tagged *Bp*GH117 were comparatively studied with those of previously characterized 3,6-anhydro-α-(1,3)-L-galactosidases, and His-tagged *Bp*GH117 was investigated to determine whether this enzyme is optimal for the human gut environment. The results of this study can be used to utilize His-tagged *Bp*GH117 for industrial use.

## 2. Results

### 2.1. Analysis of the Enzymatic Reaction Products by Thin-Layer Chromatography (TLC) and High-Performance Liquid Chromatography (HPLC)

**Figure 1:**
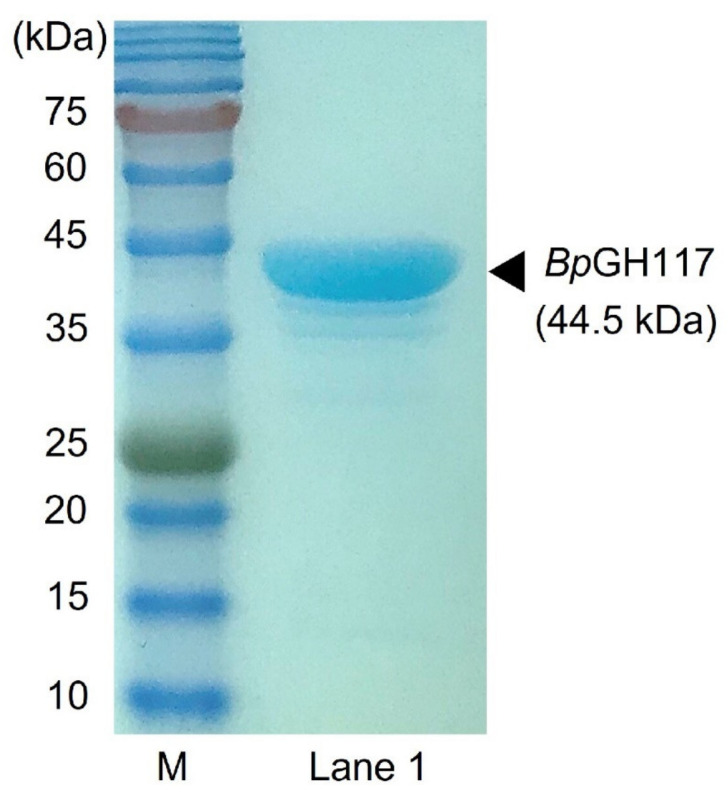
Sodium dodecyl sulfate-polyacrylamide gel electrophoresis (SDS-PAGE) analysis of purified His-tagged *Bp*GH117. Lanes: M, protein marker; Lane 1, purified His-tagged *Bp*GH117.

**Figure 2:**
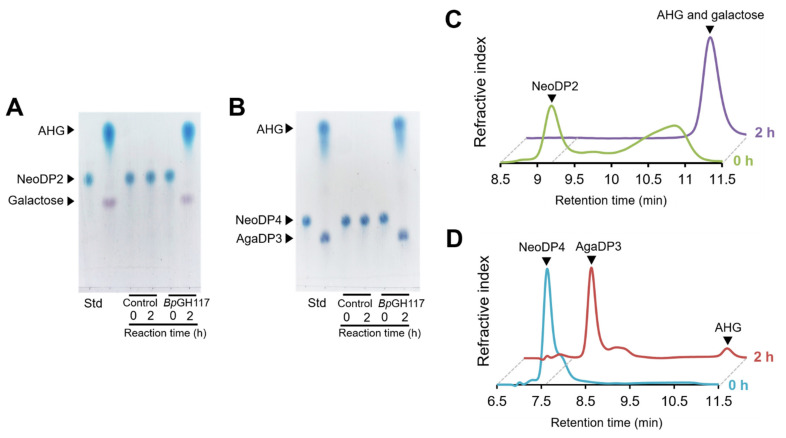
Product analyses of His-tagged *Bp*GH117 with neoagarobiose (NeoDP2) and neoagarotetraose (NeoDP4) by (**A**,**B**) thin-layer chromatography and (**C**,**D**) overlaid high-performance liquid chromatography. All reactions were carried out with 2 mg/mL NeoDP2 or NeoDP4 in 50 mM Tris-HCl (pH 9.0) buffer at 35 °C. Control: negative control containing the same volume of 50 mM Tris-HCl buffer (pH 9.0) instead of *Bp*GH117. AgaDP3, agarotriose; AHG, 3,6-anhydro-L-galactose; Std, standard.

To reveal the mode of enzymatic action of *Bp*GH117, the purified His-tagged *Bp*GH117 was prepared to react with NeoDP2 and NeoDP4. The His-tagged *Bp*GH117 overexpressed without a signal sequence was identified by sodium dodecyl sulfate–polyacrylamide gel electrophoresis (SDS-PAGE) using a theoretical molar mass of 44.5 kDa (Figure 1). The reaction products formed after the treatment of NeoDP2 or NeoDP4 with His-tagged *Bp*GH117 were analyzed by TLC and HPLC (Figure 2). First, the products formed after the treatment of NAOs, NeoDP2 and NeoDP4, with His-tagged *Bp*GH117, were visualized by TLC. According to the TLC analysis results, NeoDP2 was hydrolyzed into AHG and galactose, and NeoDP4 was hydrolyzed into AgaDP3 and AHG by enzymatic reactions with His-tagged *Bp*GH117, respectively, while NeoDP2 and NeoDP4 remained due to the negative control reaction (Figure 2A,B).

In addition, the enzymatic reaction mixtures of His-tagged *Bp*GH117 were analyzed using HPLC. When NeoDP2 was used as the substrate, a peak corresponding to NeoDP2 disappeared, while a peak corresponding to galactose and AHG appeared after 2 h reaction with His-tagged *Bp*GH117 (Figure 2C). Similarly, when using NeoDP4 as the substrate, a peak corresponding to NeoDP4 disappeared, and peaks corresponding to AgaDP3 and AHG appeared after the His-tagged *Bp*GH117 enzymatic reaction (Figure 2D). These results confirmed that *Bp*GH117 is an α-NAOH that can cleave α-1,3-glycosidic bonds in both NeoDP2 and NeoDP4.

### 2.2. Optimal pH and Temperature of BpGH117

**Figure 3:**
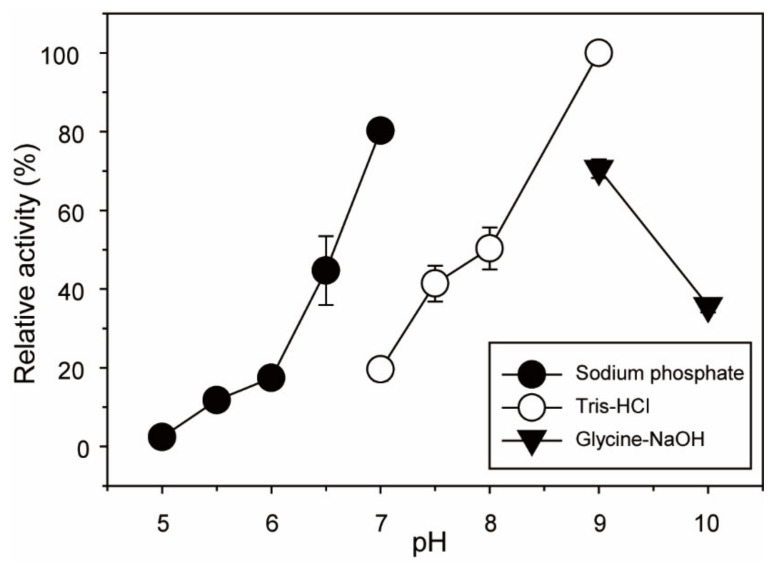
Effect of pH on His-tagged *Bp*GH117 activity. To assess the effect of pH, the reactions were performed at 35 °C for 10 min in different buffers: 50 mM sodium citrate buffer (pH 4.0), 50 mM sodium phosphate buffer (pH 5.0–7.0), 50 mM Tris-HCl buffer (pH 7.0–9.0), and 50 mM glycine-NaOH buffer (pH 9.0–10.0).

To determine the optimal pH and temperature for the enzymatic reaction of His-tagged *Bp*GH117, the enzymatic reactions were performed at various pH values and temperatures. First, the effect of pH on His-tagged *Bp*GH117 activity was evaluated by performing enzymatic reactions at pH 4.0–10.0 (Figure 3). His-tagged *Bp*GH117 showed the highest enzymatic activity at pH 9.0. Additionally, 50% of the maximum activity was maintained at pH 8.0, and 44, 41, and 36% of the maximum activity was maintained at pH 6.5, 7.5, and 10.0, respectively.

**Figure 4:**
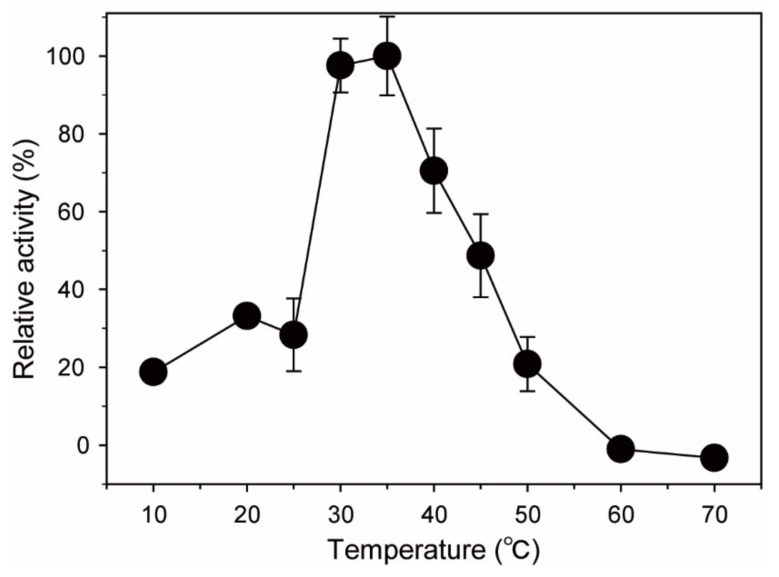
Effect of temperature on His-tagged *Bp*GH117 activity. To determine the optimal temperature of His-tagged *Bp*GH117, the reactions were performed at 10–70 °C in 50 mM Tris-HCl buffer at pH 9.0 for 10 min.

Similarly, the effect of temperature on His-tagged *Bp*GH117 activity was determined by measuring the enzyme activities at 10–70 °C (Figure 4). The highest activity of His-tagged *Bp*GH117 was observed at 35 °C. In addition, 97, 70, and 48% of the maximal activity were maintained at 30, 40, and 45 °C, respectively. However, the relative activity of His-tagged *Bp*GH117 decreased below 33% at ≤25 °C, and also decreased below 20% at ≥50 °C.

### 2.3. Thermostability of BpGH117

**Figure 5:**
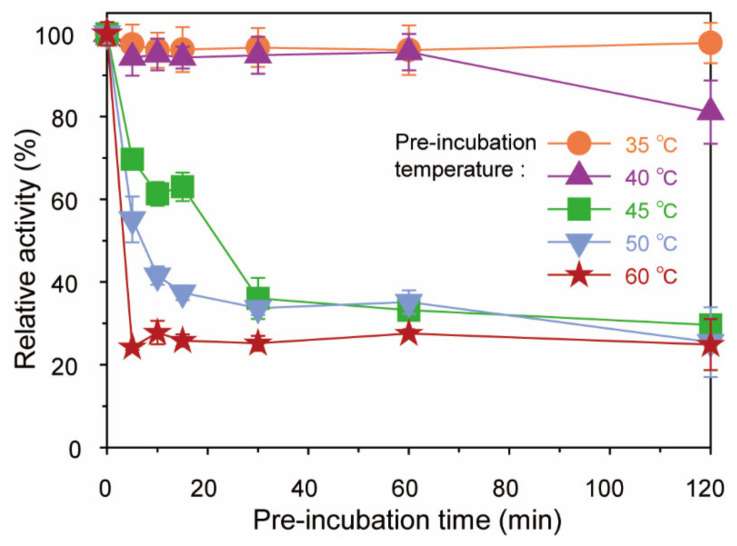
Thermostability of His-tagged *Bp*GH117. To determine the thermostability of His-tagged *Bp*GH117, His-tagged *Bp*GH117 was pre-incubated at 35–60 °C for 0–120 min before the enzymatic reaction at 35 °C for 10 min.

To determine the thermostability of His-tagged *Bp*GH117, the enzyme was pre-incubated at 35–60 °C for 0–120 min (Figure 5) before reacting with 2 mg/mL NeoDP4 in 50 mM Tris-HCl buffer (pH 9.0) at 35 °C for 10 min. His-tagged *Bp*GH117 maintained 100% of its initial activity for up to 120 min at 35 °C. Even though the residual relative activity of His-tagged *Bp*GH117 slightly decreased, more than 80% of its initial activity was maintained after pre-incubating for 120 min at 40 °C. However, the enzymatic activity after pre-incubating for 120 min at 45 °C or higher was only about 25% of the initial activity.

### 2.4. Effect of Metal Ions and EDTA on the Activity of BpGH117

The effect of various metal ions and a chelating agent, EDTA, on the enzymatic activity of His-tagged *Bp*GH117, was tested by measuring the enzyme activity in reaction mixtures containing 1 mM of the metal ions in the form of chloride salts or EDTA. The results revealed that His-tagged *Bp*GH117 activity was not affected by any metal ions tested in this study or EDTA (Table 1).

### 2.5. Kinetic Parameters of BpGH117

The kinetic parameters of His-tagged *Bp*GH117 toward NeoDP2 and NeoDP4 were determined from the Lineweaver–Burk plot. The *K_m_*, *V_max_*, and *k_cat_* values of His-tagged *Bp*GH117 toward NeoDP2 were 30.22 mM, 54.84 U/mg, and 80.1 s^−1^, respectively, while those toward NeoDP4 were 14.16 mM, 26.98 U/mg, and 40 s^−1^, respectively. Therefore, His-tagged *Bp*GH117 showed a lower *K_m_* value toward NeoDP4 than NeoDP2, which implies that His-tagged *Bp*GH117 may exhibit a higher substrate affinity toward NeoDP4 than toward NeoDP2.

The kinetic parameters, *K_m_* and *V_max_* values of His-tagged *Bp*GH117, were also compared with those of previously characterized α-NABH and α-NAOH toward NeoDP2 (Table 2). His-tagged *Bp*GH117 had the highest *K_m_* value among the characterized α-NABH and α-NAOH enzymes. In addition, His-tagged *Bp*GH117 had the fourth highest *V_max_* value among the 14 enzymes listed in Table 2.

### 2.6. Amino Acid Sequence Analysis of BpGH117

**Figure 6:**
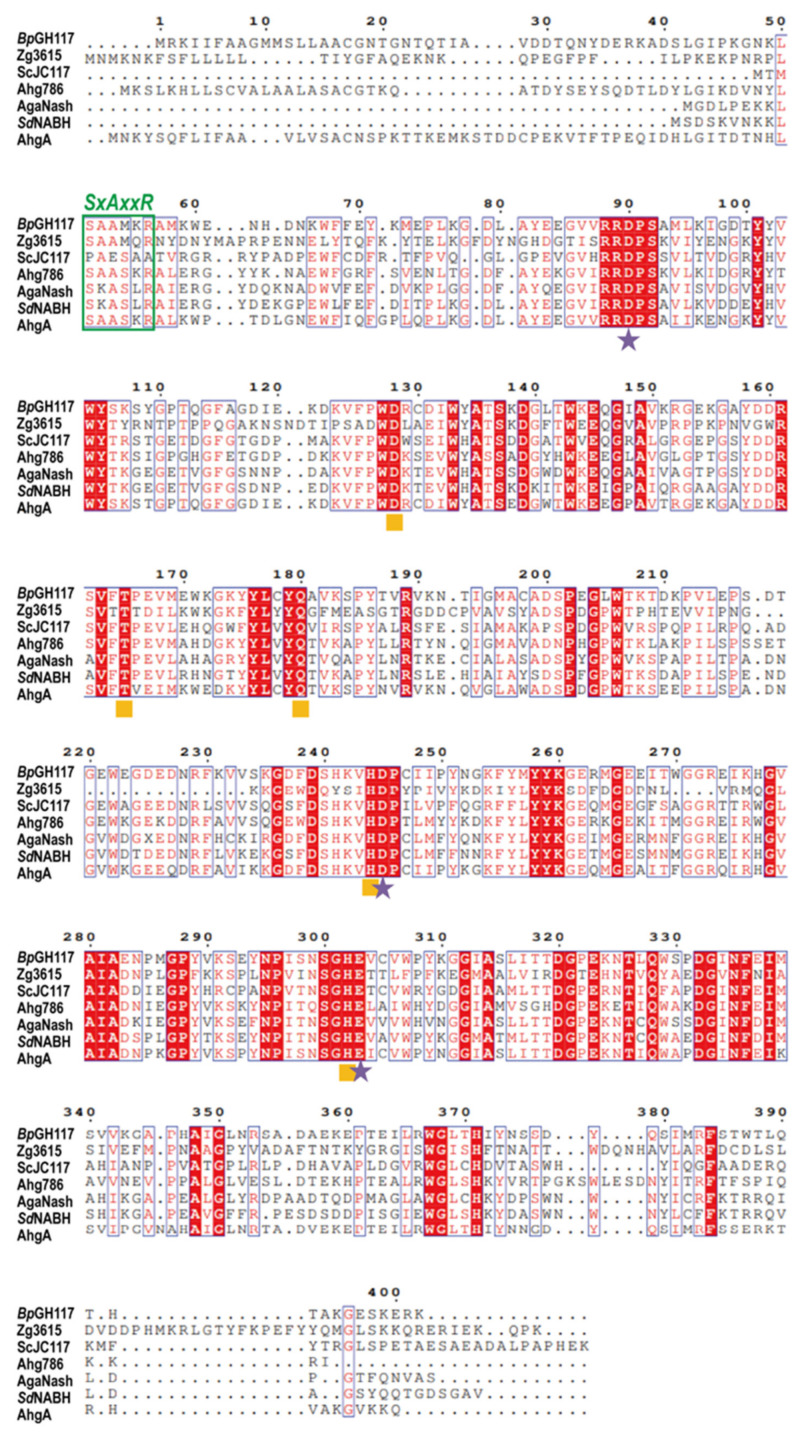
Amino acid alignment of *Bp*GH117 with other GH117 family members. Stars (★) denote catalytic residues and squares (■) indicate residues involved in substrate binding.

The BACPLE_01671 gene has 1206 base pairs and is translated into a 402-amino acid protein, *Bp*GH117. A BLAST search for available sequence databases suggested that the amino acid sequence of *Bp*GH117 was quite similar to that of several GH117 enzymes known to exhibit α-NABH or α-NAOH activity [25]. Protein sequence alignment of *Bp*GH117 showed several domains that were highly conserved with other known GH117 enzymes (Figure 6). *Bp*GH117 carries the SxAxxR motif, the signature motif of the GH117 family, which represents the basal requirement for the multimerization of GH117 enzymes and is known to be present in several GH117 enzymes [25,29,30]. The acidic amino acids Asp-90, Asp-245, and Glu-303 are probably involved in the coordination with an NAO substrate [19]. The conserved residues Trp-128, Thr-165, Gln-180, His-244, and His-302 are assumed to act as the catalytic sites of GH117 enzymes [29,30].

## 3. Discussion

α-NAOH has been suggested to play an important role in breaking down agar, a non-digestible carbohydrate [19]. Most agarolytic microorganisms are known to be marine microorganisms [7,8,9]. However, a human gut bacterium, *B. plebeius*, was recently found to have enzymes that can hydrolyze agar [10,11,12]. Agarooligosaccharides have been shown to promote the growth of beneficial strains in the intestine, suggesting their possibility as prebiotics [11]. Thus, by studying enzymes derived from the human gut bacterium, it becomes possible to further understand the processes or enzymes which decompose agarose in the intestine, and how prebiotics would be produced from agarose. Additionally, through this information, the GH117 enzyme *Bp*GH117, could be applied to a wider variety of fields, such as producing prebiotics derived from marine macroalgae. Therefore, it is important to study *B. plebeius*-derived enzymes to understand how agarose, which is usually not degraded by innate enzymes in humans, is metabolized in the intestine. However, only crystallographic studies have been performed on the *Bp*GH117 enzyme and its biochemical characteristics have been partially studied to date [19]. Thus, we characterized the enzymatic properties of His-tagged *Bp*GH117, an α-NAOH isolated from *B. plebeius*.

In this study, His-tagged *Bp*GH117 was found to be alkaline α-NAOH and α-NABH, which showed the highest activity at pH 9.0 (Figure 3). It is noteworthy that His-tagged *Bp*GH117 has optimal activity in an alkaline environment, unlike most 3,6-anhydro-α-(1,3)-L-galactosidases which showed optimal activity in a neutral environment (pH 6.0–8.0) (Table 2). The highest activity of His-tagged *Bp*GH117 was observed at 35 °C, which is similar to human body temperature, while most GH117 enzymes except *Sd*NABH exhibited the maximum activity below 30 °C (Figure 4 and Table 2). These results are attributed to the origin of His-tagged *Bp*GH117, which is *B. plebeius* isolated from the human gut.

Regarding cofactors, α-NAOH and α-NABH enzymes do not have a common metal ion requirement (Table 2). For example, ScJC117, Ahg786, *Sd*NABH, and neoagarobiose hydrolase from *Cytophaga flevensis* were inhibited by Zn^2+^ [18,23,29,33]. Ahg558, Ahg786, α-NAOH from *Cellvibrio* sp. WU-0601, *Sd*NABH, and α-NAOH from *Bacillus* sp. MK03 were inhibited by Cu^2+^ [18,24,26,29,31]. Crystallographic data for *Bp*GH117 showed that the protein binds to Mg^2+^ ions [19]. However, it was revealed that metal ions do not affect His-tagged *Bp*GH117 activity in vitro.

To date, the *k_cat_/K_m_* values of α-NABH and α-NAOH toward NeoDP2 have been reported for only four enzymes, most of them, except Ahg558, being less than 1 s^−1^ mM^−1^, whereas *k_cat_/K_m_* of His-tagged *Bp*GH117 was 2.65 s^−1^/mM [23,24,26,27]. The high *k_cat_/K_m_* value of *Bp*GH117 suggests that the enzyme has high catalytic efficiency. This implies that His-tagged *Bp*GH117 may hydrolyze NAOs, including NeoDP4 and NeoDP2, more efficiently than most other GH117 enzymes.

In this study, His-tagged *Bp*GH117 was found to cleave the α-1,3-glycosidic linkage from the non-reducing ends of NAOs, including NeoDP2 and NeoDP4. More specifically, when His-tagged *Bp*GH117 hydrolyzes NeoDP4, AgaDP3 and AHG are produced. Odd-numbered agarooligosaccharides have been reported to have prebiotic effects by showing that probiotic strains, *B. infantis* and *B. adolescentis*, decompose AgaDP3 and grow with AgaDP3 as the sole carbon source [11]. In addition, AHG has been known to have various physiological activities such as anti-inflammatory, skin whitening, and anticariogenic activities [21,22]. Therefore, His-tagged *Bp*GH117 enzyme would be advantageous in producing high value-added products such as AHG and AgaDP3 from agarose, owing to its higher *k_cat_/K_m_* value than most other α-NABH and α-NAOH enzymes. In conclusion, *Bp*GH117 originating from *B. plebeius* was characterized as a GH117 enzyme from human gut bacterium in this study. In particular, His-tagged *Bp*GH117 derived from human gut bacterium has unique optimal conditions for enzymatic activity at 35 °C and pH 9.0. Furthermore, His-tagged *Bp*GH117 showed the second highest *k_cat_/K_m_* value toward NeoDP2 among the characterized GH117 enzymes. Notably, His-tagged *Bp*GH117 can produce value-added products including AgaDP3 and AHG when NAOs such as NeoDP2 and NeoDP4 are given as a substrate. Therefore, *Bp*GH117 can be used to produce bioactive agar-derived products, and information about its optimal enzymatic reaction conditions revealed in this study can also be utilized for its industrial processes.

## 4. Materials and Methods

### 4.1. Overexpression and Purification of Recombinant BpGH117

The gene BACPLE_01671 encoding *Bp*GH117 without a signal sequence (1–54 bp) was cloned into the pET-21α(+) vector (Novagen, Madison, WI, USA), and the recombinant plasmid was transformed into *Escherichia coli* BL21(DE3) (Novagen). To produce recombinant His-tagged *Bp*GH117, recombinant *E. coli* BL21(DE3) harboring the *Bp*GH117 gene was incubated in Luria–Bertani (LB; BD; San Jose, CA, USA) broth medium containing 100 µg/mL of ampicillin (Sigma-Aldrich, St. Louis, MO, USA) at 37 °C until the culture reached the mid-exponential phase of growth.

When the optical density at 600 nm (OD_600_) reached 0.5, 0.5 mM isopropyl-β-D-1-thiogalactopyranoside (IPTG; Sigma-Aldrich, St. Louis, MO, USA) was added to the culture medium to induce recombinant His-tagged *Bp*GH117. After incubation for 16 h at 16 °C, the cells were harvested by centrifugation at 10,000 × *g* for 30 min at 4 °C. The cell pellet was resuspended in ice-cold lysis buffer (20 mM Tris-HCl, pH 7.4) and the cell suspension was disrupted using a sonicator (Branson, Gunpo, Korea). The supernatant containing the soluble protein was collected by centrifugation at 15,000 × *g* for 40 min at 4 °C. The recombinant His-tagged *Bp*GH117 was purified by affinity chromatography using a His-Trap column (GE Healthcare, Piscataway, NJ, USA) and the eluent buffer containing 0.5 M NaCl and 0.1 M imidazole in 20 mM sodium phosphate buffer (pH 7.4). The purified His-tagged *Bp*GH117 was concentrated using an Amicon ultrafiltration membrane (MW cutoff 30 kDa; Millipore, Billerica, MA, USA). The protein concentration was determined using a bicinchoninic acid (BCA) protein assay kit (Thermo Fisher Scientific, Waltham, MA, USA).

### 4.2. Enzyme Activity Measurement Using 3,5-Dinitrosalicylic Acid (DNS) Assay

The enzyme activity of His-tagged *Bp*GH117 was determined by measuring the amount of released reducing sugar in the reaction mixture using the DNS method with D-galactose as a monomeric sugar standard [35]. To prepare NeoDP2 and NeoDP4 as the substrates for reactions by His-tagged *Bp*GH117, we carried out the enzymatic degradation of agarose, followed by purification. For the degradation of agarose, two in-house recombinant enzymes were used: an endo-type β-agarase, *Bp*GH16A, which produces NeoDP4 as the major product from agarose [36], and an exo-type β-agarase, Aga50D, which produces NeoDP2 from agarose. NeoDP2 and NeoDP4 were purified from each reaction product by gel filtration chromatography using Bio-Gel P-2 Gel polyacrylamide (Bio-Rad, Hercules, CA, USA) and distilled water as an eluent. The enzymatic reaction mixture containing 0.05 mg/mL recombinant His-tagged *Bp*GH117 and 2 mg/mL NeoDP2 or NeoDP4 in 50 mM Tris-HCl buffer (pH 9.0) was incubated at 35 °C for 10 min. As a negative control, the same volume of 50 mM Tris-HCl buffer (pH 9.0) was incubated instead of the enzyme. The reaction mixture was incubated in boiling water for 5 min to terminate the enzymatic reaction. To determine the amount of total reducing sugar produced, 60 µL of the DNS solution was added to 60 µL of the enzymatic reaction mixture. The mixture was incubated at 95 °C for 5 min and cooled at 4 °C for 5 min. The absorbance at 540 nm was recorded using a microplate spectrophotometer (xMark; Bio-Rad, Hercules, CA, USA) to measure the concentration of reducing sugars. One unit (U) of *Bp*GH117 activity was defined as the amount of enzyme required to release 1 µmol of reducing sugar per minute under the above reaction conditions.

### 4.3. TLC and HPLC Analyses of Enzymatic Reaction Products

For analyzing the products generated after the substrate was completely reacted, the enzymatic reaction was performed for 2 h under the same conditions as when the DNS analysis was performed. First, the products formed by treating NeoDP2 or NeoDP4 with His-tagged *Bp*GH117 were analyzed by TLC. An aliquot of 1 µL from each reaction sample was spotted on silica gel 60 TLC plates (Merck, Darmstadt, Germany), which were developed with water: ethanol: *n*-butanol (1:1: 3, *v/v*). The plates loaded with samples were visualized by spraying 10% (v/v) H_2_SO_4_ in ethanol and 0.2% (w/v) naphthoresorcinol in ethanol [21]. The reaction products were also analyzed by HPLC (Agilent Technologies, Santa Clara, CA, USA) system with an Aminex HPX-87H column (Bio-Rad) and a refractive index detector (Agilent Technologies). HPLC analysis was performed at 65 °C using 0.005 N H_2_SO_4_ as the mobile phase at a flow rate of 0.5 mL/min.

### 4.4. Biochemical Characterization of BpGH117

The optimal pH of His-tagged *Bp*GH117 activity was determined by incubating 0.05 mg/mL His-tagged *Bp*GH117 with 2 mg/mL NeoDP4 at 35 °C for 10 min at pH 4.0–10.0 using different buffers, depending on the pH: 50 mM sodium citrate buffer for pH 4.0, 50 mM sodium phosphate buffer for pH 5.0–7.0, 50 mM Tris-HCl buffer for pH 7.0–9.0, and 50 mM glycine-NaOH buffer for pH 9.0–10.0. To determine the optimal temperature of His-tagged *Bp*GH117 activity, 0.05 mg/mL His-tagged *Bp*GH117 was incubated with 2 mg/mL NeoDP4 in 50 mM Tris-HCl buffer (pH 9.0) for 10 min at 10–70 °C.

To measure the thermostability of His-tagged *Bp*GH117, prior to the enzymatic reaction, 0.05 mg/mL His-tagged *Bp*GH117 in 50 mM Tris-HCl buffer (pH 9.0) was pre-incubated at 30–70 °C for 0–120 min. After pre-incubation, the enzymatic reaction was performed by adding 2 mg/mL NeoDP4 to the pre-incubated mixture and incubating at 35 °C for 10 min. After pre-incubation, residual enzyme activity was determined, and His-tagged *Bp*GH117 activity without pre-incubation was considered as 100%.

To study the effect of metal ions and a chelating agent, EDTA, on His-tagged *Bp*GH117 activity, various metal ions in the form of chloride salts, Na^+^, K^+^, NH_4_^+^, Li^+^, Ca^2+^, Mg^2+^, Mn^2+^, and Rb^2+^, and EDTA were used. The enzymatic reaction was performed by incubating 0.05 mg/mL His-tagged *Bp*GH117 with 2 mg/mL NeoDP4 in 50 mM Tris-HCl buffer (pH 9.0) containing 1 mM of each ion or EDTA at 35 °C for 10 min. *Bp*GH117 activity measured in the absence of metal ions or EDTA was considered to be 100%.

### 4.5. Determination of the Kinetic Parameters of BpGH117

The kinetic parameters of His-tagged *Bp*GH117 were determined by the enzymatic reactions of 0.05 mg/mL His-tagged *Bp*GH117 with 0.5–4 mg/mL NeoDP2 or NeoDP4 at 35 °C in 50 mM Tris-HCl buffer (pH 9.0) for 10 min. The *V_max_, K_m_,* and *k_cat_* values were calculated from the Lineweaver–Burk plot based on the Michaelis–Menten kinetics (Figure S1 in the Supplementary Materials) [37].

### 4.6. Amino Acid Sequence Analysis of BpGH117 for Comparison with other GH117 Enzymes

The amino acid sequence of *Bp*GH117 was compared using the BLAST program of the National Center for Biotechnology Information (NCBI; https://blast.ncbi.nlm.nih.gov/Blast.cgi, accessed on 12 June 2020) and UniProt (http://www.uniprot.org/blast/, accessed on: 12 June 2020). Espript and Clustal omega were used for multiple sequence alignment of the *Bp*GH117 amino acid sequence [38,39].

## Figures and Tables

**Table 1:** **Effect of metal ions and EDTA on His-tagged *Bp*GH117 activity.** The enzyme activity was determined with various metal ions in the form of chloride salts or EDTA at the final concentration of 1 mM. The enzyme activity without metal ions or EDTA was considered as 100%.

	Relative Activity (%)
Control	100.0 ± 4.3
**Metal ion in the form of chloride salt**	
KCl	92.6 ± 11.6
NaCl	102.7 ± 8.7
NH_4_Cl	98.8 ± 5.6
LiCl	95.5 ± 12.7
CaCl_2_	80.0 ± 4.2
MgCl_2_	100.3 ± 7.4
RbCl_2_	100.9 ± 7.9
MnCl_2_	98.6 ± 7.9
**Chelating agent**	
EDTA	98.1 ± 0.7

**Table 2:** **Comparison of characterized α-neoagarobiose/neoagarooligosaccharide hydrolases.** *K_m_* and *V_max_* values are toward neoagarobiose (NeoDP2). n.a., not available, Identity (%), a number that describes how similar the query sequence is to the target sequence (how many characters in each sequence are identical).

Strain (Enzyme)	Molar Mass of Subunit (kDa)	Monomer/ Multimer	Location of Protein	Effect of Metal Ion		Optimum		*K_m_* (mM)	*V_max_* (U/mg)	Substrate	Identity (%)	Reference
				Activation	Inhibition	Temp. (°C)	pH					
*Bacteroides plebeius* (*Bp*GH117)	45.6	Dimer	Extracellular	n.a.	n.a.	35	9.0	30.22	54.84	NeoDP2/4/6		This study, [19]
*Streptomyces coelicolor* A3(2) (ScJC117)	41	n.a.	Extracellular	Mg^2+^	Ba^2+^, Ca^2+^, Co^2+^, Fe^3+^, Zn^2+^, Ni^2+^	30	6.0	11.57	n.a.	NeoDP2/4/6	51.8	[23]
*Gayadomonas joobiniege* G7 (Ahg558)	40.8	Dimer	n.a.	Mn^2+^	Cu^2+^, Mg^2+^	30	9.0	8.01	133.33	NeoDP2/4/6	59.9	[24]
*Gayadomonas joobiniege* (Ahg786)	45.18	Dimer	Extracellular	Mn^2+^	Cu^2+^, Mg^2+^, Zn^2+^, Ni^2+^	15	7.0	4.5	1.33	NeoDP2/4/6	56.9	[18]
*Cellulophaga* sp. W5C (AhgI)	45	Octamer	Extracellular	Ca^2+^	n.a.	20–30	7.0	1.03	10.22	NeoDP2/4/6	68.1	[25]
*Cellvibrio* sp. WU-0601	42	Dimer	Cytosolic	Mn^2+^, Mg^2+^	Ag^+^, Hg^2+^, Cu^2+^, Ni^2+^	25	6.0	5.8	60	NeoDP2/4/6	58.5	[26]
*Agarivorans gilvus* WH0801 (AgaWH117)	41	n.a.	Cytosolic	n.a.	n.a.	30	6.0	6.45	6.98	NeoDP2/4	59.0	[27]
*Cellvibrio* sp. OA-2007	40	Dimer	Cytosolic	n.a.	n.a.	32	7.0–7.2	6	19	NeoDP2/4/6	57.0	[28]
*Saccharophagus degradans* 2–40^T^ (*Sd*NABH)	41.6	Dimer	Cytosolic	n.a.	Zn^2+^, Ni^2+^, Cu^2+^, Co^2+^	42	6.5	3.5	n.a.	NeoDP2/4/6	60.3	[29]
*Zobellia galactanivorans* (AhgA)	41	Dimer	Extracellular	n.a.	n.a.	n.a.	n.a.	n.a.	n.a.	NeoDP4/6	69.1	[30]
*Bacillus* sp. MK03	42	Octamer	Extracellular	Mg^2+^	Ag^+^, Ni^2+^, Cu^2+^, Hg^2+^	30	6.1	n.a.	22.2	NeoDP2/4/6	n.a.	[31]
*Vibrio* sp. JT0107	42	Dimer	Cytosolic	n.a.	n.a.	30	7.7	5.37	92	NeoDP2/4/6	n.a.	[32]
*Cytophaga flevensis*	n.a.	n.a.	Cytosolic	n.a.	Ag^+^, Hg^2+^, Zn^2+^, Pb^2+^	25	6.75	n.a.	n.a.	NeoDP2	n.a	[33]
*Pseudomonas atlantica*	10	n.a.	Periplasmic	Na^+^	n.a.	n.a.	7.3–8.0	n.a.	n.a.	NeoDP2	n.a	[34]

## Data Availability

The datasets used and/or analyzed during the current study are available from the corresponding author upon reasonable request.

## Supplementary Materials

The following are available online at https://www.mdpi.com/article/10.3390/md19050271/s1, Figure S1: Lineweaver-Burk plot of *Bp*GH117.
