# Supplementary material for: Characterization of Neoagarooligosaccharide Hydrolase BpGH117 from a Human Gut Bacterium Bacteroides plebeius
Source: Mar Drugs. 2021 May 13;19(5):271. doi: 10.3390/md19050271 (PMC8152962; doi:10.3390/md19050271)
Supplement: Supplementary file 1 [file marinedrugs-19-00271-s001.zip › marinedrugs-1148129-supplementary.pdf]

Supplementary Materials

Characterization of neoagarooligosaccharide hydrolase *Bp*GH117 from a human gut bacterium *Bacteroides plebeius*

Yerin Jin†, Sora Yu†, Dong Hyun Kim, Eun Ju Yun, and Kyoung Heon Kim*

Department of Biotechnology, Graduate School, Korea University, Seoul 02841, South Korea

*Correspondence: khekim@korea.ac.kr

†These authors contributed equally to this work.

To obtain the kinetic parameters of *Bp*GH117, the enzymatic reactions were performed using different concentrations of neoagarobiose (NeoDP2) ranging from 0.5 to 4 mg/ml in the total reaction mixture volume 1 ml containing 0.05 mg/ml *Bp*GH117 at a pH of 9.0 and 35°C for 10 min. The values of K_m and V_{max} were calculated from the Lineweaver-Burk plot. One unit (U) of *Bp*GH117 activity was defined as the amount of enzyme required to release 1 μmol of reducing sugar per min, in which reducing sugar was quantified by the dinitrosalicylic acid method using galactose as the sugar standard.

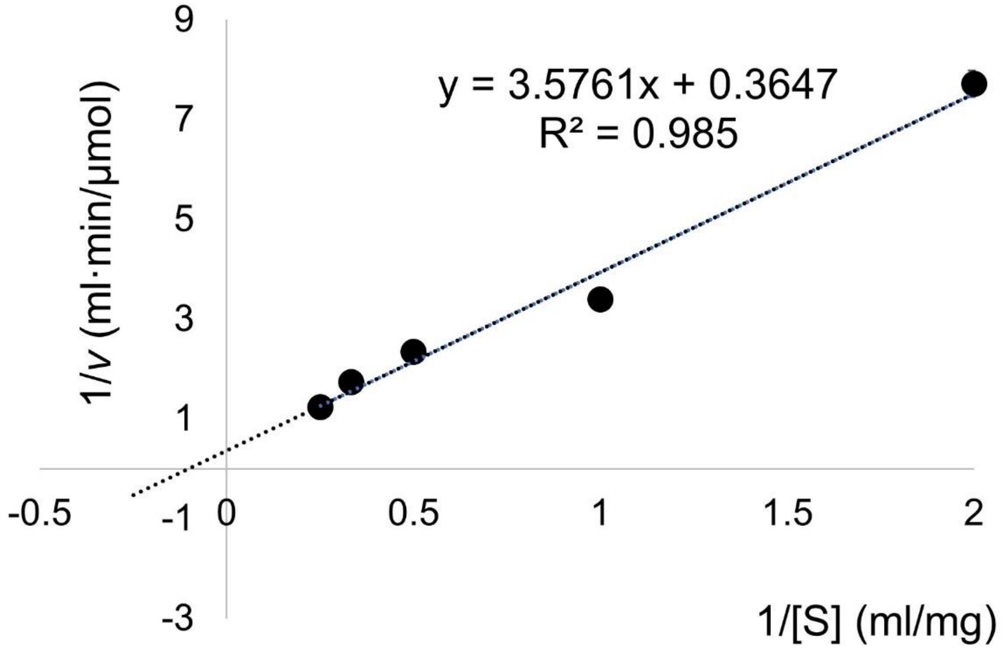

Figure S1 Lineweaver-Burk plot of *Bp*GH117

To determine the kinetic parameters, initial velocity experiments were performed, and the Michaelis-Menten equation (S1.1) was used. From the experiment, the product

concentrations were plotted against time to determine the initial velocity (v) with various initial substrate concentrations at the fixed initial concentration of *Bp*GH117 (i.e., $[E_o]$ = 0.05 mg/ml).

$$v = \frac{d[P]}{dt} = \frac{-d[S]}{dt} = k_{cat} \qquad (S1.1)$$

$$v = \frac{V_{max}[S]}{K_m + [S]} \qquad (S1.1a)$$

$$\frac{1}{v} = \frac{-1}{V_{max}} + \frac{K_m}{V_{max}} \frac{1}{[S]} \qquad (S1.1b)$$

Using the initial velocities and the initial substrate concentrations, the Lineweaver-Burk plot was obtained (Fig. S1). A plot of 1/v versus 1/[S] yielded a straight line with a slope, K_m/V_{max} and a y-axis intercept, $1/V_{max}$. In the Lineweaver-Burk plot, when y was 0, x was $-1/K_m$.

$$V_{max} = k_{cat}[E_0] \qquad (S1.2)$$

The K_m and V_{max} values of *Bp*GH117 on NeoDP2 as the substrate were 9.8 mg/ml and 2.7 µmol/(ml/min), respectively. From the definition of U of *Bp*GH117, 2.7 µmol/(ml/min) = 2.7 U/ml. To alternatively express V_{max} in U/mg protein, 2.7 U/ml of V_{max} was divided by 0.05 mg/ml, and V_{max} was expressed as 54.84 U/mg protein.

To determine k_{cat}, the turnover number, Equation S1.2 was used. The molar

mass of recombinant His-tagged *Bp*GH117 in monomeric form is 44.5 kDa, but the *Bp*GH117 can function only in dimeric form as the minimal unit of the functioning enzyme. Therefore, in determining k_{cat}, 1 µmol of *Bp*GH117 was considered (2 x 44.5 kDa) x 10^{-6} mol = 89.0 mg, and the initial enzyme concentration, $[E_0]$, was 0.05 mg/ml = 5.62 x 10^{-4} µmol/ml. The value of k_{cat} was calculated to be 80.1 s^{-1} as shown in Equation S1.3.

$$k_{cat} = \frac{2.7\ \mu\text{mol/(ml/min)}}{5.62 \times 10^{-4}\ \mu\text{mol/ml}} \times \frac{\text{min}}{60\ \text{s}} = 80.1\ \text{s}^{-1} \qquad \text{(S1.3)}$$

Finally, using the values of k_{cat} and K_m, the catalytic efficiency of *Bp*GH117, k_{cat}/K_m, was estimated to be 8.17 s^{-1}/(mg/ml). Since the MW of NeoDP2 is 324.28, k_{cat}/K_m was also expressed as 2.65 s^{-1}/mM.
